# Copper Deficiency as Wilson’s Disease Overtreatment: A Systematic Review

**DOI:** 10.3390/diagnostics13142424

**Published:** 2023-07-20

**Authors:** Tomasz Litwin, Agnieszka Antos, Jan Bembenek, Adam Przybyłkowski, Iwona Kurkowska-Jastrzębska, Marta Skowrońska, Anna Członkowska

**Affiliations:** 1 Second Department of Neurology, Institute of Psychiatry and Neurology, 02-957 Warsaw, Poland; agantos@ipin.edu.pl (A.A.); ikurkowska@ipin.edu.pl (I.K.-J.); mskowronska@ipin.edu.pl (M.S.); czlonkow@ipin.edu.pl (A.C.); 2 Department of Clinical Neurophysiology, Institute of Psychiatry and Neurology, 02-957 Warsaw, Poland; jbembenek@ipin.edu.pl; 3 Department of Gastroenterology and Internal Medicine, Medical University of Warsaw, 02-091 Warsaw, Poland; aprzybylkowski@interia.pl

**Keywords:** Wilson’s disease, copper, neuropathy, myelopathy, pancytopenia

## Abstract

Background: Treatment of Wilson’s disease (WD), an inherited disease characterized by copper overload, is lifelong and there is the possibility that copper deficiency (CD) may occur. We systematically reviewed the literature to describe treatment patterns, symptoms and outcomes associated with CD. Methods: Using preferred reporting items for systematic reviews and meta-analyses (PRISMA) guidelines, the PubMed database was searched up to 6 April 2023. Results: Across 17 articles, 20 cases of CD were described, most commonly (15 cases) in WD patients treated with zinc salts (ZS), less often on combined chelator and ZS therapy (3 cases), molybdate salts plus ZS (1), or molybdate alone (1). CD symptoms occurred insidiously, including sideroblastic anemia, neutropenia, axonal sensory neuropathy, posterior cord myelopathy and increased ratio of epileptic seizures (or epilepsy). CD diagnosis was based on symptoms and severely reduced urinary copper excretion (<20 µg/24 h [<0.3 µmol/24 h] on ZS, or <100 µg/24 h [<1.6 µmol/24 h] on chelators) with low total serum copper and ceruloplasmin. Conclusions: Awareness of CD and regular monitoring of copper metabolism is needed during WD treatment. Temporary cessation of anti-copper treatment usually reverses serum copper reductions as well as pancytopenia; however, some symptoms, especially neuropathy and myelopathy, may persist.

## 1. Introduction

Wilson’s disease (WD) is an inherited disorder of copper metabolism with systemic copper overload, leading to clinical symptoms in affected organs, mainly hepatic and neuropsychiatric [1,2,3]. The pathogenesis of WD includes dysfunction of trans-membrane copper transporting type P ATPase 7B, involved in copper excretion from hepatocytes into the bile and copper incorporation into ceruloplasmin (Cp) [1,2,3]. Failure of this transporting protein leads to progressive copper accumulation in hepatocytes, their progressive damage and release of bioavailable so-called “free” non-Cp-bound copper (NCC) into the blood with secondary copper accumulation, and damage to other organs and tissues. In the course of WD, high serum NCC is observed, usually above 15 µg/dL (normal range: 5–15 µg/dL), in contrast to total serum copper which decreases below 70 µg/dL (normal range: 70–140 µg/dL) in line with decreased serum Cp level [1,2].

NCC has not been validated as a reliable biomarker of WD treatment due to the fact that the current method for NCC calculation is indirect (calculated from the difference between the copper concentrations in μg/dL and 3.15 times the serum Cp concentration in mg/dL) and may give non-diagnostic false negative results in up to 20% of patients, particularly when immunological methods of Cp assessment are used due to overestimation of holoceruloplasmin. Direct methods to measure NCC are under investigation [2,4]. The most promising is direct quantification of copper pools from human plasma by combining Cp immunocapture, chelation and filtered inductively coupled plasma mass spectrometry (ICP-MS) to permit quantitative analysis of Cp-copper and directly measured NCC; however, this method currently requires validation before introduction into WD management [2,4,5].

As WD is caused by copper body overload and intoxication, pharmacological treatment is based on drugs, leading to negative systemic copper balance. Chelators (d-penicillamine [DPA] and trientine [TN]) increase urinary copper excretion, while zinc salts (ZS) decrease copper absorption from the digestive tract, and currently in clinical trials, molybdenum salts (tetrathiomolybdate [TH]) have been shown to chelate excess copper and thereby reduce copper levels by biliary excretion and decrease copper absorption from the digestive tract [1,2,3,6,7,8,9,10,11].

Correct anti-copper treatment should lead to the normalization of calculated, indirect NCC (5–15 µg/dL); the stabilization and increase of copper urinary copper excretion between 200–500 µg/day in the case of chelators; and a decrease of <100 µg/day in the case of ZS use, as well as clinical symptoms regression [1,2]. However, in some cases, especially following long-term treatment, copper deficiency (CD) may occur [1,2,9].

Copper is an essential important cofactor for many enzymes (e.g., cytochrome-C oxidase, dopamine-beta-hydroxylase, copper-zinc superoxide dismutase, proline hydroxylase, lysine oxidase and ascorbic oxidase) involved in the functioning of the bone marrow (erythropoiesis), the central nervous system (CNS; myelinization, dopamine synthesis), skin and vessel construction (collagen synthesis and bridging), as well as respiratory chain enzymes (mitochondrial and other energetic processes). Hence, CD due to WD overtreatment may lead to severe multisystem symptoms including hematological (bone marrow dysfunction leading to anemia and neutropenia) and neurological disorders (e.g., axonal neuropathy, myelopathy, posterior spinal column demyelination and myopathy) [10,11,12,13,14,15,16,17,18,19,20,21,22,23,24,25,26,27,28,29,30,31].

As the frequency of CD in WD, its association with this type of anti-copper treatment, its risk factors and clinical outcomes have not been analyzed, we performed a systematic review of published studies and case reports on CD in WD.

## 2. Methods

This systematic review was performed in concordance with the internationally accepted criteria of the preferred reporting items for systematic reviews and meta-analyses (PRISMA) statement [32].

### 2.1. Literature Search and Eligibility Criteria

By searching the PubMed database (up to 6 April 2023), we identified eligible original studies (prospective and retrospective), as well as case and series reports describing and analyzing patients with WD and CD. Terms used for searches included: “copper deficiency and Wilson’s disease/Wilson disease” and “copper depletion and Wilson’s disease/Wilson disease.” Eligible studies were defined as: (1) human studies; (2) original studies (prospective or retrospective); (3) case and series reports of CD in WD patients; (4) published in English. The reference lists of extracted publications were also searched. 

Titles and abstracts of all retrieved papers were screened independently by all authors and duplicate records were removed. Reviews, editorials, commentaries, and overlapping studies were excluded after assessment and revisions. The full texts of eligible articles were checked according to eligibility. All identified studies were analyzed and verified independently by all authors to confirm the inclusion criteria and analyze the data. 

The study protocol was registered and received INPLASY registration number: Inplasy protocol 202340004. Available online: https://doi.org/10.37766/inplasy2023.4.0004 (accessed on 10 July 2023).

### 2.2. Data Extraction

The following data were extracted from eligible studies: (1) study characteristic: authors, year of publication, study design (prospective, retrospective, series report or case report); (2) patient characteristic—gender, WD phenotype, age at WD diagnosis and CD onset, initial anti-copper treatment, copper metabolism at CD diagnosis; (3) CD clinical symptoms with available examinations which confirmed diagnosis; (4) CD treatment and outcome. The data extraction was conducted by all authors, with any disagreements being resolved by consensus.

### 2.3. Statistical Analysis

The statistical analysis were carried out using Statistica v.10 (Stat Soft Inc. 2011, Tulsa, OK, USA). The mean, range, percentage, and standard deviation (SD) were noted for descriptive summary statistics.

## 3. Results

**Figure 1:**
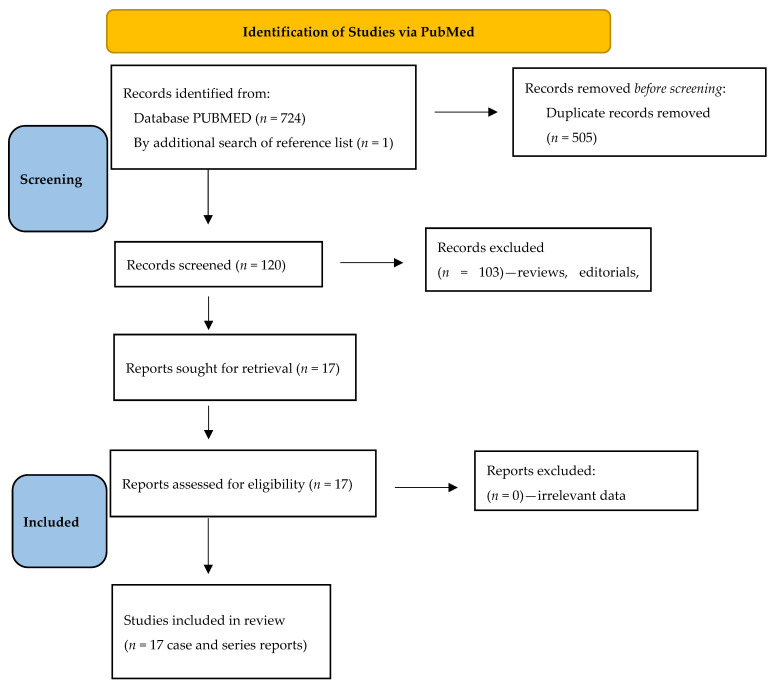
Flow chart of the systematic literature search according to PRISMA guidelines.

During the systematic review of the literature, we found 725 records from PubMed and reference list searches (Figure 1). After duplicate paper removals, 120 papers remained. Further analyzing titles, abstracts and full texts, we additionally removed 103 records. Finally, 16 papers [10,11,12,13,14,15,16,17,18,19,20,21,22,23,24,29] and 1 abstract [28] were included in the analysis, describing 20 WD patients with CD as case and series reports (Table 1).

In the included papers, there was equal distribution of cases according to gender (10 male and 10 female). Mean age of CD occurrence was 34.9 ± 15.0 years (range: 12–57) and mean duration of WD treatment before CD diagnosis was 15.7 ± 11.7 years (range: 1–38). Mean Cp was 2.53 ± 2.0 mg/dL and serum total copper was 8.04 ± 8.0 µg/dL; mean urinary copper excretion was 14.4 ± 10.2 µg/day on ZS and 93 ± 53.0 µg/day on chelators with ZS (Table 1). Among the 20 cases described, CD most commonly occurred in WD patients treated with ZS (15/20; 75%), less frequently on combined therapy with chelators and ZS (3/20; 15%), molybdate salts (TH) and ZS (1/20; 5%) or on TH alone (1/20; 5%). Most frequent clinical symptoms of CD included severe sideroblastic anemia (11/20; 55%), leukopenia (9/20; 45%), neutropenia (11/20; 55%), sensory axonal neuropathy (7/20; 35%), posterior cord myelopathy (6/20; 30%); increased ratio of epileptic seizures (or epilepsy) (2/20; 10%), white matter demyelination (1/20; 5%).

Management of CD included temporary anti-copper drug cessation in all cases, temporary copper supplementation in 5 cases, blood transfusions in 2 cases (due to severe anemia), and treatment with granulocyte stimulating factor in 1 case (Table 1). Hematological parameters (hemoglobin, red blood cells, white blood cells and neutrophils) returned to normal values in all cases. Neurological deficits resulting from polyneuropathy and myelopathy subsided in only 1 case (16%) and improved partially in a further 5 cases (83%). Reduction of epileptic seizures was observed in both cases.

## 4. Discussion

Based on our literature searches, theoretical mechanisms and a general review, the clinical symptoms of CD in patients with WD, as in the population as a whole [29,30], include predominantly hematological changes (especially anemia [sideroblastic] with leuko/neutropenia) and neurological-like sensorimotor distal polyneuropathy and spinal cord myelopathy (cervical or thoracic), and rarely, white matter demyelination and epileptic seizures.

The molecular basis of anemia is likely due to the fact that copper is a component of key enzymes involved in iron hemostasis. Cp has ferroxidase activity that converts ferrous iron to ferric iron, to enable iron incorporation into apo-transferrin and transport iron in the blood. Hence, Cp defects, such as aceruloplasminemia, can lead to iron accumulation in macrophages, anemia and neurodegeneration. Hephaestin is another copper-containing protein with ferroxidase activity, which is involved in iron release from intestinal cells. As with Cp, the lack of transformation from ferrous to ferric ions decreases the release of iron from enterocytes, leading to iron deficiency and anemia. Finally, copper-dependent cytochrome C in mitochondria is involved in reducing ferric into ferrous ions, which is required for iron incorporation in hemoglobin synthesis. Animal studies also indicate that copper-deficient rats have decreased erythrocyte survival, probably due to oxidative stress from a lack of antioxidant copper-dependent cytochrome C activity, leading to damaged membranes and increased vulnerability [29,30,31,33,34].

Interactions between copper and iron resulting in hematopoiesis disturbances were first described in 1932 [34]. In animal models, researchers observed reduced iron uptake in rats fed with a copper-deficient diet and impaired utilization of parenterally administered iron for hemoglobin synthesis in copper-deficient swine, which led to anemia with low hemoglobin levels as well as cytopenia. Based on this observation, without knowledge about copper-dependent proteins with ferroxidase activity, copper-related anemia terms were used in the bibliography [30,34].

The etiology of other hematological symptoms includes the destruction of myeloid progenitor cells, copper-dependent impaired maturation of myeloid precursors, as well as the formation of anti-neutrophils, antibodies related to copper depletion. However, in contrast to anemia, the exact mechanism of these phenomenon is not known. Analyzing the hematological profile, only thrombocytopenia is not in the spectrum of CD symptoms, and the finding of normal platelet values with severe anemia and neutropenia in patients where CD is suspected is an argument in favor of a CD diagnosis [30,31].

Neurological symptoms including myeloneuropathy [12,13,15,16,17,18,19,20,27], CNS demyelination [11] and epileptic seizures [14,28] are also observed in copper-deficient syndromes due to Menkes disease, in which a lack of activity of copper-dependent enzymes involved in CNS maturation lead to these symptoms. The exact mechanism(s) of CD-related myeloneuropathy are unknown [12,13,15,16,17,18,19,20,27]. Myelopathy affecting the spinal cord (cervical or thoracic) seems to be most common neurological presentation of CD syndrome. Clinically and radiologically, it is not possible to distinguish between subacute cord degeneration due to vitamin B12 deficiency and CD. However, CD is suspected as the cause of cytochrome C (copper-dependent) dysfunction, with increased lactate found in cerebrospinal fluid in contrast to vitamin B12-dependent cord degeneration. Another hypothesis for CD-related myelopathy is the dysfunction of methylation cycles, including myelin proteins (with spinal cord damage) and neuropathy.

CD symptoms appeared to occur after long-term anti-copper treatment, and most commonly in those treated with ZS or ZS plus chelators. The causality of CD is probably caused by differences in monitoring the anti-copper adequacy of treatment in WD. Urinary copper excretion should be less than 100 µg/24 h with ZS and 200–500 µg/24 h with chelators. Hence, it is easier to overlook decreased urinary copper excretion in CD when patients are treated with ZS than with chelators, especially in long-term treated WD patients when decreased Cp and serum copper are observed. Based on the increasing numbers of CD in WD, current American Association for the Study of Liver Diseases (AASLD) recommendations state that CD may be suspected when daily urinary copper excretion is low: <20 µg/24 h (<0.3 µmol/24 h) in patients treated with ZS or <100 µg/24 h (<1.6 µmol/24 h) on chelators and associated with low serum total copper and low Cp; however, cut-offs for serum copper and Cp are not provided [2]. As TH is currently under investigation in clinical trials, this treatment is not discussed in WD guidelines [1,2,3]. However, based on its dual mechanism of action, low daily copper urinary excretion with low Cp and serum copper, as in ZS, may arouse suspicion of CD.

Analyzing CD outcomes, in the course of anti-copper treatment dose reduction or temporary cessation, hematological symptoms disappeared in all cases over a relatively short period of time, although blood transfusion was needed in some severe cases of anemia. However, while there was some improvement in neurological symptoms with WD treatment discontinuation, they persisted in most patients, which underlines the need for correct and timely diagnosis of CD in WD.

In summarizing, the results from our study highlight the significance of monitoring copper metabolism as well as hematological parameters in WD patients to detect CD, especially those treated for a long duration. Furthermore, from the neurologist’s point of view, the occurrence of sensory disturbances in WD patients as well as gait deterioration should always include differential diagnosis of CD, especially in ZS-treated patients, as these symptoms could be potentially reversible [35,36]. However, improper increase of anti-copper medications doses due to suspicion of WD progression may lead to irreversible neurological complications.

Our study has several limitations. Firstly, our searches only retrieved case reports and the total number of patients included was small; however, there were no prospective studies and just 20 case reports in the published literature. Furthermore, the lack of data from retrospective studies did not allow us to establish the frequency of CD in WD and follow-up observations were described over a limited period of time. We did not perform a meta-analysis, as study designs and available data came only from case reports (no prospective or retrospective studies in WD patients analyzing these problems with statistical analysis have been published so far). Furthermore, available data were heterogeneous (including copper metabolism biomarkers, as well as clinical data).

In addition, analysis of Cp was conducted using different methods (immunologic and enzymatic assay), which did not allow us to calculate NCC across the whole group of patients [4,5].

Currently, in order to improve the copper metabolism monitoring, additional copper metabolism biomarkers are studied which include directly measured NCC, labile bound copper (LBC), exchangeable copper (CuEXC) and relative exchange copper (REC) (last both used mostly in France, especially in WD diagnosis) [4,5]. They need to be validated in WD treatment monitoring, as well as in centers involved in WD management. The establishment of the objective, direct measurement of copper metabolism biomarkers will allow us to avoid more frequent CD syndromes by using more adequate doses of anti-copper drugs and more detailed copper metabolism monitoring [1]. Finally, another issue that may improve the monitoring of WD treatment (including adverse events) are the prospective international registries of WD patients. This may help to establish the frequency of CD and other adverse drug reactions in the course of WD treatment, by providing more reliable data. Only such data from studies will allow for meta-analyses for risk factors of CD, and therefore better characterize this problem [1,5].

## 5. Conclusions

Physicians treating WD patients should be aware of and familiar with CD symptoms, which include mostly anemia with neutropenia and myeloneuropathy. The diagnosis of CD is based mainly on clinical symptoms and copper metabolism, especially low daily copper urinary excretion. CD usually occurs in patients treated long-term, particularly with ZS. Early detection of CD and temporary anti-copper treatment cessation (or dose decrease) leads to rapid improvement of hematological parameters. Neurological symptoms may subside over a longer time window, but may persist in the case of long-lasting CD.

## Figures and Tables

**Table 1:** Summary of case reports analyzing copper deficiency in patients with Wilson’s disease.

Reference	Patient	Details of WD	Symptoms and Signs of CD	CD Treatment	Outcome
Ueda et al., 2022 [24]	57-year-old man with neurological WD diagnosed aged 20	Initially on DPA 1000 mg/day and ZA 80 mg/day; ZA increased to 150 mg/day due to neurological progression (dysphagia with gastrostomy) Cu metabolism at CD diagnosis: Cp: 5.5 mg/dL Serum Cu: 11 µg/dL Urinary Cu excretion: 74 µg/24 h	Moderate distal weakness, hypotonia, absent tendon reflexes, sensory ataxia and severe sensory disturbances in all 4 limbs (numbness and paresthesia) Severe sensory motor polyneuropathy (EDX) Myelopathy in cervical MRI in posterior cord Anemia (Hgb 6.9 g/dL)	ZA and DPA cessation for 9 months, with increased Cu intake to 1.67 mg/day DPA introduced thereafter	Partial recovery after 3 months Dysesthesias reduced; about 40% improvement in gait MRI changes in spinal cord persisted Anemia stopped progressing (Hgb 9.3 g/dL after 3 weeks and 10.8 g/dL after 3 months) Cu metabolism: N/A
Wu et al., 2020 [20]	18-year-old woman with neurological WD diagnosed aged 17	Initial 1 year on DPA and TN but stopped due to ADR(skin rash after DPA, abdominal pain after TN). ZS initiated at 150 mg/day, increased to 225 mg/day Cu metabolism at CD diagnosis: Cp and serum Cu: N/A Urinary Cu excretion: “low”	Distal weakness of both legs, sensory disturbances of legs, falls Axonal sensory neuropathy (EDX) and abnormal SSEPs Myelopathy in thoracic MRI in posterior cord Hematology N/A	ZS cessation with Cu supplementation 10 mg/day for 6 months	Complete recovery after 6 months Complete resolution of neuropathy and myelopathy (no follow-up MRI provided) Cu metabolism: N/A
Cai et al., 2019 [23]	12-year-old female with presymptomatic WD diagnosed aged 7 (abnormal LFTs that normalized during WD treatment)	ZG (150 mg elemental zinc), increased to 240 mg at age 8 due to initially increased LFT Cu metabolism at CD diagnosis: Cp: N/A Serum Cu: 12.7 µg/dL Urinary Cu excretion: 30 µg/24 h	Abnormal gait (no details) Brain MRI normal, spine MRI rejected by patient Anemia (Hgb 4.0 g/dL; RBC 1.37 M/µL), leukopenia (WBC 1.5 K/µL) and neutropenia (0.08 K/µL)	Temporary ZG cessation, blood transfusion	Complete recovery Gait disturbance subsided Hgb started to increase after 1 week, with normalization after 8 months (Hgb 11.7 g/dL, RBC 5.28 M/µL) Cu metabolism: N/A
Mohamed et al., 2018 [22]	26-year-old woman with neurological WD diagnosed aged 13	ZS 200 mg/day of elemental zinc, increased to 240 mg due to initially increased LFT Cu metabolism at CD diagnosis: Cp: 2 mg/dL Serum Cu: <0.1 µmol/L Urinary Cu excretion: N/A	Bone marrow biopsy: moderate dyserythropoiesis with defects in maturation Spine MRI and SSEPs not undertaken Anemia (as above), leukopenia (WBC 1.2 K/µL) and neutropenia (0.2 K/µL)	Temporary ZS cessation and supplementation Cu gluconate 2 mg/day After 8 months, ZS at 50% dose planned	Complete recovery Hgb normalization after 4 months (Hgb 12.2 g/dL, neutrophil 2.5 K/µL) Serum Cu 1 µmol/L at 8 months
Dzieżyc et al., 2014 [19]	37-year-old asymptomatic woman diagnosed aged 21 (family screening)	16 years on ZS 180 mg/day Cu metabolism at CD diagnosis: Cp: 0.92 mg/dL Serum Cu: <5 µg/dL Urinary Cu excretion: 11 µg/24 h	Paresthesias in fingers and toes for 3 months, weakness in lower limbs during fast walking Posterior column of the cervical cord (C2-Th1) myelopathy in spine MRI Myelopathy (few low-amplitude short-duration motor unit [EDX]) Leukopenia (WBC 2.9 K/µL)	ZS cessation for 9 months, DPA introduced later	CD symptoms disappeared after 6 months EDZ normalization; SSEPs abnormalities remained in dorsal column Improved WBC (6.0 K/µL) Cp: 5 mg/dL Serum Cu: 20 µg/dL Urinary Cu excretion: 12.5 µg/24 h
	46-year-old man with hepatic WD diagnosed aged 41 (compensated liver failure)	5 years on ZS (180 mg/day) Cu metabolism at CD diagnosis: Cp: 0.5 mg/dL Serum Cu: <5 µg/dL Urinary Cu excretion: 6 µg/24 h	Upper respiratory tract infections Leukopenia (WBC 1.86 K/µL) Neutropenia (neutrophils 0.89 × 10^9^/L)	Decreased ZS dose to 135 mg/day and further treat WD with decreased dose of ZS (with close follow-up)	Complete recovery after 12 months Improved hematology (WBC 3.3 K/µL; neutrophils 2.0 × 10^9^/L) Cp: 1.18 mg/dL Serum Cu: 5 µg/dL Urinary Cu excretion: 10.5 µg/24 h
	18-year-old woman with presymptomatic WD diagnosed aged 12 (family screening)	6 years on ZS (180 mg/day) Cu metabolism at CD diagnosis: Cp: 0.9 mg/dL Serum Cu: 7 µg/dL Urinary Cu excretion: 12 µg/24 h	Anemia (RBC 3.2 M/µL) Leukopenia (WBC 2.3 K/µL) Neutropenia (neutrophils 0.17 × 10^9^/L) SSEPs: impaired conduction in spinal dorsal column (refused MRI)	Cessation of ZS for 2 months initially (later for longer 1-year period)	Complete recovery after 1 year Improved hematology (WBC 7.3 K/µL; neutrophils: 5.1 × 10^9^/L) Cp: 17 mg/dL Serum Cu: 44 µg/dL Urinary Cu excretion: 10 µg/24 h
Rau et al., 2014 [21]	16-year-old male diagnosed aged 14 (form not provided)	1 year on DPA then switched to ZS (dose not described) due to anemia and neutropenia Cu metabolism at CD diagnosis: Cp: <2 mg/dL Serum Cu: 31 µg/dL Urinary Cu excretion: 32 µg/24 h	Anemia (Hgb 5.8 g/dL; RBC 1.9 M/µL) Leukopenia (WBC 1.4 × 10^9^) Neutropenia (0.46 × 10^9^/L) SSEPs, EDX, MRI of spine not undertaken	Cessation of ZS for 1 year, Cu supplementation 2.5 mg/day for a few months, DPA introduced later slowly	Complete recovery after 8 weeks: Improved hematology (Hgb 13.6 g/dL, RBC 5.3 M/µL, WBC 6.3 K/µL, neutrophils 3.23 × 10^9^/L Serum Cu: 63 µg/dL Cp and urinary Cu excretion: N/A
Kaleagasi et al., 2013 [28]	29-year-old patient (details not described)	Treatment with DPA and ZA (dose not known) Cu metabolism at CD diagnosis: Serum Cu: 0.3 µg/dL Cp and urinary Cu excretion: N/A	Recurrent seizures, complex partial seizures and secondary generalized seizures Hematology, SSEPs and MRI of head and spine not undertaken	ZA and DPA stopped and low Cu diet introduced	Complete recovery Seizures frequency decreased Serum Cu: 16 µg/dL Cp and urinary Cu excretion: N/A
Teodoro et al., 2013 [18]	36-year-old man with neurological WD diagnosed aged 20	16 years on ZS 150 mg/day and TN 500 mg/day (few years on DPA 600 mg/day instead of TN due to lack of TN complicated by nephrotic syndrome, then TN and ZS 330 mg/day) Cu metabolism at CD diagnosis: Cp: 3 mg/dL Serum Cu: 13.3 µg/dL Urinary Cu excretion: 41 µg/24 h	Numbness of both hands and feet and gait worsening with falls Posterior dorsal cord myelopathy (in spine MRI) Mixed sensory/motor peripheral neuropathy (EDX) Hematology and SSEPs not undertaken	TN and ZS were substituted by ZA 100 mg/day, which was progressively reduced and stopped due to persistent CD After 1 year ZA 150 mg/day reintroduced	Partial improvement at 1 year Improvement in gait without support, improvement in electrophysiological assessment of neuropathy Myelopathy decreased in MRI Cp and serum Cu: N/A Urinary Cu excretion: 97 µg/24 h
Lozano Herrero et al., 2012 [17]	56-year-old woman with hepatic WD diagnosed aged 18	28 years with DPA 750 mg/day, last 10 years on ZA 503 mg (150 mg elemental zinc) Cu metabolism at CD diagnosis: Cp: undetectable Serum Cu: 3 µg/dL Urinary Cu excretion: undetectable	Slowly progressive unstable gait with paresthesia of hands and feet Myelopathy in cervical cord C2-C7 in MRI EDX normal, abnormal SSEPs Anemia (Hgb 11.3 g/dL) Neutropenia (neutrophils 0.7 × 10^9^/L)	ZA cessation and Cu supplementation (later ZA slowly introduced at low dose)	Minimal improvement during first few months Cp: 12.3 mg/dL Serum Cu: 36 µg/dL Urinary Cu excretion: 46 µg/24 h
Cortese et al., 2011 [15]	51-year-old woman with neurological WD diagnosed aged 19	32 years of treatment, initially on DPA, then switched to ZS 600 mg/day (elemental zinc unknown) due to hyperintense reaction polyadenopathy Cu metabolism at CD diagnosis: Cp: N/A Serum Cu: 5 µg/dL Urinary Cu excretion: 20 µg/24 h	Distal limb paresthesia, sensory loss, and gait disturbances Sensory motor peripheral polyneuropathy (EDX) SSEPs and MRI of spinal cord normal Anemia (Hgb 6.5 g/dL) Neutropenia (neutrophils 0.25 × 10^9^/L)	ZS reduction to 600 mg/day initially and substituted by ZA 150 mg/day (not data according to elemental zinc) and blood transfusions	Improvement in cytopenia and anemia without effect on neuropathy Cp: N/A Serum Cu: 64 µg/dL Urinary Cu excretion: 42 µg/24 h
Da Silva-Junior et al., 2011 [16]	44-year-old woman with neurological WD diagnosed aged 29 (complete resolution of neurological symptoms in 1 year)	15 years of treatment, initially on DPA but stopped due to ADR, then on ZA 450 mg/day (elemental zinc not known) Cu metabolism at CD diagnosis: Cp: 8 mg/dL Serum Cu: 3 µg/dL Urinary Cu excretion: 7.4 µg/24 h	Progressive numbness of feet, then also arms Peripheral sensory neuropathy Spinal cord myelopathy C1-C6 Leukopenia (WBC 2.7 × 10^9^/L)	ZA cessation	Symptoms stabilized (partial improvement) 4 months later Serum Cu: 37 µg/dL CP and urinary Cu excretion: N/A
Benbir et al., 2010 [14]	21-year-old man with neurological WD diagnosed aged 16	5 years on DPA 1200 mg/day and ZA 100 mg/day (180 mg/day) Cu metabolism at CD diagnosis: Cp: N/A Serum Cu: 2.2 µg/dL Urinary Cu excretion: 165 µg/24 h	Status epilepticus with partial seizures Epileptiform activity on EEG Lack of hematology results	Cessation of DPA and ZA, high Cu diet (discharged home on ZA only) Anti-convulsant treatment (levetiracetam and diazepam)	Complete seizures did not re-occur Serum Cu: 13.7 µg/dL CP and urinary Cu excretion: N/A
Horvath et al., 2010 [13]	41-year-old man diagnosed with neurological WD aged 25	On DPA but stopped after 6 months due to ADR, then on ZS (200 mg elemental zinc) for 14 years; increased to 245 mg for 1 year due to fatigue and agitation (CD symptoms) Cu metabolism at CD diagnosis: Cp: 2 g/dL Serum Cu: 0.1 µmol/L Urinary Cu excretion: 0.4 µmol/24 h	Gait disturbances, fatigue Anemia (Hgb 7.8 g/dL) Leukopenia (WBC 2.1 × 10^9^/L) Neutropenia (neutrophils 0.26 × 10^9^/L) Axonal sensory peripheral polyneuropathy with distal amyotrophy (EDX) Abnormal SSEPs MRI of spine normal	Cessation of ZS	Complete resolution of hematological changes in 10 months Polyneuropathy persisted at 1 year follow-up Cp: 0.02 g/L Serum Cu: 0.27 µmol/L Urinary Cu excretion: 0.35 µmol/24 h
Foubert-Samier et al., 2009 [12]	43-year-old man diagnosed with neurological WD aged 15	18 years on TN up to 900 mg/day and ZA 400 mg/day (elemental zinc not known) Cu metabolism at CD diagnosis: Cp: <1 mg/dL Serum Cu: 0.5 µmol/L Urinary Cu excretion: 1.7 µmol/24 h	Distal limb weakness Sensory motor peripheral axonal neuropathy (EDX) Anemia (Hgb 10.6 g/dL) Neutropenia (neutrophils 0.95 × 10^9^/L)	Cessation of ZA, and decreased TN dose (300 mg/day) then increased to 900 mg/day	Anemia and neutropenia disappeared Neuropathy persisted at 2-year follow-up Cu metabolism: N/A
Narayan et al., 2006 [11]	13-year-old male with neurological WD diagnosed aged 9	4 years on DPA 750 mg/day and ZS 280 mg/day (elemental zinc not known) Cu metabolism at CD diagnosis: Cp: 2.34 mg/dL Serum Cu: 16 µg/dL Urinary Cu excretion: N/A	Sensory disturbances in legs (decreased vibratory on joints) Anemia (no results provided) Demyelination in brain CT (white matter tracts)	Not provided	Not provided
Karunajeewa et al., 1998 [29]	44-year-old man with neurological WD diagnosed aged 21	Initially on DPA 1000 mg/day, reduced to 500 mg/day due to leukopenia after 6 months. Then on TN for 2 years, switched to DPA with ZS for 11 years. Switched to TH 200 mg/day and ZS 440 mg/day (elemental zinc not known) Cu metabolism at CD diagnosis: Cp: 2 mg/dL Serum Cu: 2 µmol/L Urinary Cu excretion: 0.1 µmol/24 h	Anemia (Hgb 50 g/dL) Leukopenia (WBC 1 K/µL) Neutropenia (0.36 × 10^9^/L) Bone marrow biopsy showed marked hypoplasia	ZS and TH cessation for 9 months, filgrastim commenced, neutropenia disappeared, then reintroduction of TH 50 mg/day after 8 months	After 4 weeks: improved hematology (Hgb 11.6 g/dL; WBC 4.4 K/µL) Bone marrow biopsy showed mild hypocellularity and left shifted granulopoiesis Cu metabolism: N/A
Karunajeewa et al., 1998 [29]	34-year-old woman with hepatic WD diagnosed aged 17	On DPA 2000 mg/day initially. After splenectomy, increased to 2500 mg/day for 8 years due to excessive Cu liver deposition; switched to TH 200 mg/day Cu metabolism: N/A	Anemia (Hgb 7.1 g/dL) Neutropenia (neutrophils 0.35 × 10^9^/L) Low Cu levels in liver biopsy: 46 µg/g	TH cessation for 4 weeks, reintroduced at lower dose of 50 mg/day	After 4 weeks: improved hematology (Hgb 11.5 g/dL, neutrophils 4.9 × 10^9^/L) Cu metabolism: N/A
Van Den Hamer & Hoogenrad 1989 [10]	56-year-old patient diagnosed aged 25	31 years of treatment, initially on DPA then switched to ZS 1200 mg/day (elemental zinc not known). CD occurred 2 years after switch Cu metabolism at CD diagnosis: Serum Cu: 0.05 µg/mL Cp and urinary Cu excretion: N/A	Anemia with neutropenia	Parenteral Cu supplementation	Complete recovery Normalization of hematology in a few days Cu metabolism: N/A

ADR = adverse drug reaction; CD = copper deficiency; Cp = ceruloplasmin; Cu = copper; DPA = d-penicillamine; EDX = electrodiagnostic testing, including nerve conduction studies and needle electromyography; EEG = electroencephalogram; Hgb = serum hemoglobin; LFT = liver function test; MRI = magnetic resonance imaging; N/A = not available; RBC = red blood cells; SSEPs = somatosensory evoked potentials; TN = trientine; TH = tetrathiomolybdate; WBC = white blood cells; WD = Wilson’s disease; ZA = zinc acetate; ZG = zinc gluconate; ZS = zinc sulphate.

## Data Availability

Not applicable.
